# Virtual Hospital, Real Health Care

**DOI:** 10.1016/j.mcpdig.2025.100303

**Published:** 2025-10-27

**Authors:** Tejaswini Manavi, Derek O’Keeffe

**Affiliations:** a School of Medicine, Health Innovation Via Engineering (HIVE) Laboratory, Cúram Research Ireland Centre for Medical Devices, University of Galway, Galway, Ireland; b Department of Medicine, University Hospital Galway, Galway, Ireland

Health systems globally are navigating a dual crisis: rising life expectancy and a tsunami of chronic diseases such as diabetes, chronic obstructive pulmonary disease (COPD), and heart failure (HF). These conditions strain conventional hospital health care models, with overcrowded emergency departments, bed shortages, and mounting clinical burnout becoming the norm. The World Health Organization projects that deaths from noncommunicable diseases will increase by 17% by 2030,^1^ a trajectory that calls for urgent, systemic transformation. Digital health (DH) has emerged not only as a distant innovation but also as an essential enabler of resilient, efficient, and equitable health care systems.

The convergence of telemedicine, remote monitoring, mobile health applications, wearable sensors, electronic health records, and artificial intelligence–driven diagnostics is reshaping how care is delivered. Within this landscape, 2 models stand out. Hospital-at-home (HaH) delivers inpatient level care at home through multidisciplinary teams, offering a safe alternative to hospital admission. Virtual wards (VWs) combine technology-enabled remote monitoring with occasional face-to-face visits to prevent admissions and support early discharge.^2^ In practice, the terms HaH and VW are sometimes used interchangeably, although they represent distinct care models.

With that in mind, a virtual hospital can be understood as a coordinated collection of VWs, designed to deliver hospital-level care through digitally enabled and team-based services. For example, a virtual hospital may host condition-specific wards such as COPD, HF, or frailty, each managed by dedicated clinical teams under 1 hospital-led framework.

Evidence supporting these models has expanded rapidly, accelerated by COVID-19. Virtual ward and HaH programs encompass several inpatient-level care models, including low technology-enabled care delivered by hospital and community-based teams and early supported discharge programs. Most models may reduce readmissions compared with hospital-based care, although impacts on mortality are less certain, with only these 2 models showing no increased risk.^2^

The National Health Service in United Kingdom has invested heavily in scaling VWs. From fewer than 10,000 patients in 2022, the program has expanded to more than 160,000 adults and over 6400 children treated at home by early 2024, preventing nearly 9000 admissions in the South East region within a single year.^3^ In Australia, HaH services delivering intravenous therapy and rehabilitation reported emergency revisit rates of only 3%. A randomized controlled trial at Massachusetts General Hospital compared acute hospital care at home with usual inpatient care for 91 adults admitted via the emergency department. Outcomes showed that a substantial portion of acute care can be delivered at home with equivalent quality and safety, 20% reduced costs, and 20% improved patient experience, measured using Likert scales.^4^

Hospital-at-home programs for patients with COPD have been implemented globally. In Canada, the Ontario Health Technology Assessment found both early discharge and admission avoidance models to be safe, effective, although limited by resource and community-care constraints. In Spain, Hospital Clinic Barcelona’s COPD-focused HaH program uses daily multidisciplinary visits, structured escalation protocols, and remote monitoring to reduce admissions and improve patient satisfaction. In the United States, COPD-focused HaH programs show comparable outcomes with inpatient care, although scalability is constrained by program variability and fragmented reimbursement. Together, global evidence indicates COPD HaH is safe, patient centered, and effective when supported by multidisciplinary teams and health system infrastructure.^5^

The University Hospital Galway COPD Virtual Care Program adds further evidence from Europe. This study evaluated patients with confirmed COPD otherwise eligible for hospital admission. Participants received a DH kit (a pulse oximeter, blood pressure monitor, thermometer, and tablet device) for twice-daily monitoring, with data reviewed by clinicians. The program demonstrated high patient satisfaction and usability scores, with outcomes improving over longer program participation. Family support emerged as a key factor in program success,^6^ and the initiative received national recognition for innovation in health care technology.

Emerging examples from lower- and middle-income countries (LMICs) reinforce adaptability. In Brazil, a randomized trial of a multifaceted telemedicine intervention for patients with HF reduced rehospitalizations by 44% at 6 months compared with usual care, driven by earlier detection of warning signs and timely adjustment of treatment.^7^ In Singapore, a cohort of 238 patients managed in a COVID-19 VWs demonstrated a median stay of 6 of 7 days, with low escalation rates and safe outcomes, preserving inpatient capacity during pandemic surges.^8^ Integration challenges still remain in LMICs, particularly around health care infrastructure, timely follow-up, and patient engagement.

Workforce models are critical to sustainability. Virtual wards and HaHs require multidisciplinary teams—physicians, nurses, allied health professionals, and coordinators, who must adapt to new models of care delivery. A persistent challenge is layering these responsibilities onto existing clinical duties leading to workload intensification and burnout. Clinicians also face uncertainty around credentialing, role delineation, and integration of virtual responsibilities into workflows. To make these models viable, health systems must redesign workforce structures—protected time for virtual care; clear scope of practice; and structured training in remote risk assessment, escalation management, and digital communication. Supportive staffing ratios, career pathways, and continuous professional development are essential to embed these models sustainably and prevent erosion of workforce morale.

Policy and reimbursement challenges remain considerable. Many jurisdictions historically favor in-person visits in payment rules, limiting incentives for virtual care. In the United States, the Centers for Medicare & Medicaid Services (CMS) Acute Hospital Care at Home waivers introduced during COVID-19 allowed Medicare inpatient payments for home-based acute care and catalyzed rapid expansion, but long-term sustainability depends on future policy decisions. Early evaluations show positive outcomes but raise questions about payment levels, quality metrics, and cost allocation. International reviews note that bundled payments and outcomes-based models align incentives better for integrated virtual care than fee-for-service.^9^

Cybersecurity requires deliberate, system-level attention. Beyond data breach, resilient models increasingly use zero-trust architectures, multifactor authentication, and stricter access controls. Governance frameworks and clinician training are essential to address vulnerabilities from human error, legacy systems, and unprotected end points.^10^ International standards such as ISI/IEC 27701 offer guidance on privacy information management. Without such measures, public trust in virtual health initiatives risks erosion, undermining adoption.

Looking forward, priority research should focus on equitable implementation of virtual care. This includes randomized trials in diverse populations, especially in LMICs; implementation studies explicitly examining barriers, facilitators, and inequities; evaluations of reimbursement and regulation; and assessments of advanced technologies, including artificial intelligence–enabled triage, for potential biases and differential impacts. Across these efforts, integrating clinical and patient-reported outcomes will be essential to fully understand benefits and harms and to guide equitable, high-quality policies.

Facility-bound, reactive care models are increasingly inadequate. Virtual ward and HaH approaches represent a digital-first, home-based ecosystem of care that is both feasible and necessary. Realizing this potential will require systemic alignment—investment in infrastructure, workforce training, equitable policy frameworks, and robust governance. The shift is inevitable; the question is whether health systems will adapt quickly enough to meet the rising demand. The path is clear: DH is not the future of health care—it is the present.

## Potential Competing Interests

The authors report no competing interests.
